# Detection of circulating tumor cells in patients with pituitary tumors

**DOI:** 10.1186/s12885-018-4162-7

**Published:** 2018-03-27

**Authors:** Gao Hua, He Yanjiao, Liu Qian, Wang Jichao, Zhang Yazhuo

**Affiliations:** 1 Beijing Neurosurgical Insititute, Capital Medical University, Tiantan xili 6#, Beijing, 100050 China; 2 Key laboratory of central nervous system injury research, Tiantan xili 6#, Beijing, 100050 China; 3 Center of Brain Tumor, Beijing Institute for Brain Disorders, Tiantan xili 6#, Beijing, 100050 China; 4 China National Clinical Research Center for Neurological Diseases, Tiantan xili 6#, Beijing, 100050 China; 5 Department of Neurosurgery, Xinjiang Uygur Autonomous Region People’s Hospital, Xinjiang, 830000 China

**Keywords:** Pituitary adenomas, Pituitary carcinomas, Circulating tumor cells, β-catenin, Ki-67, p53

## Abstract

**Background:**

Circulating tumor cells (CTCs) are tumor cells that have shed from a primary tumor and circulate in the peripheral blood. Recent experimental and clinical studies show that CTCs can be detected in early-stage disease.

**Case presentation:**

We report three cases of pituitary adenoma (PA) in which tumor cells with particles were detected in the interstitial vascular compartment by transmission electron microscopy. Tumors were completely resected. Immunohistochemical analysis showed a β-catenin score of 10.5 ± 1.5 in the three cases with CTCs compared with 2.4 ± 0.5 in 24 control adenomas. The Ki-67 labeling index was 2.1 ± 0.7 in CTCs vs. 0.2 ± 0.3 in control cases (*p* = 0.043), and the p53 score was 4.33 ± 1.3 vs. 0.31 ± 0.17 (*p* = 0.000). The E-cadherin score did not differ significantly between the two groups.

**Conclusions:**

CTCs can be detected in benign tumors such as PAs and not only in late-stage malignant tumors with apparent distant metastases. The present findings suggest that pituitary carcinomas develop from adenomas.

## Background

Pituitary adenomas (PAs) account for 15% of intracranial neoplasms and are generally considered benign, although they exhibit a wide range of behaviors [1]. Many factors affect the proliferation of pituitary adenomas, such as angiogenesis [2], apoptosis [3], growth factors [4], oncogenes [5], tumor suppressor genes, and hormone receptors [6]. The most important predictor of recurrence in functioning adenomas is the basal postoperative hormone level, whereas in nonfunctioning adenomas, no single convincing factor could be identified. Age, gender, tumor size, and invasion are not related to recurrence [7]. The ability to predict tumor recurrence at the initial surgery would be helpful for decision-making regarding adjunctive therapy and to decrease morbidity. Pituitary carcinomas are very rare tumors and are defined as primary neoplasms of the adenohypophysis that undergo craniospinal and/or systemic spread [8]. Whether pituitary carcinomas develop from adenomas or regrowth remains unclear.

Circulating tumor cells (CTCs) are tumor cells that have shed from a primary tumor and circulate in the peripheral blood. Recent studies show that CTCs can be detected not only in late-stage malignant tumors with apparent distant metastases, but also in early-stage disease. In addition, CTCs are potentially useful clinical markers for the diagnosis of malignant tumors and for decision-making regarding treatment [9]. The enumeration of CTCs is correlated with clinical outcome in prostate cancer and has been tested and used in clinical practice [10]. CTCs have been detected after chemotherapy in inflammatory breast cancer patients at high risk for relapse [11]. In the present study, we reported three cases of PA in which tumor cells with secretory granules were detected in tumor blood vessels. The expression of β-catenin, E-Cadherin, Ki-67, and p53 was analyzed in cases and in 24 control PAs to explore the mechanisms underlying recurrence and metastasis.

## Case presentation

### Case 1

**Fig. 1:**
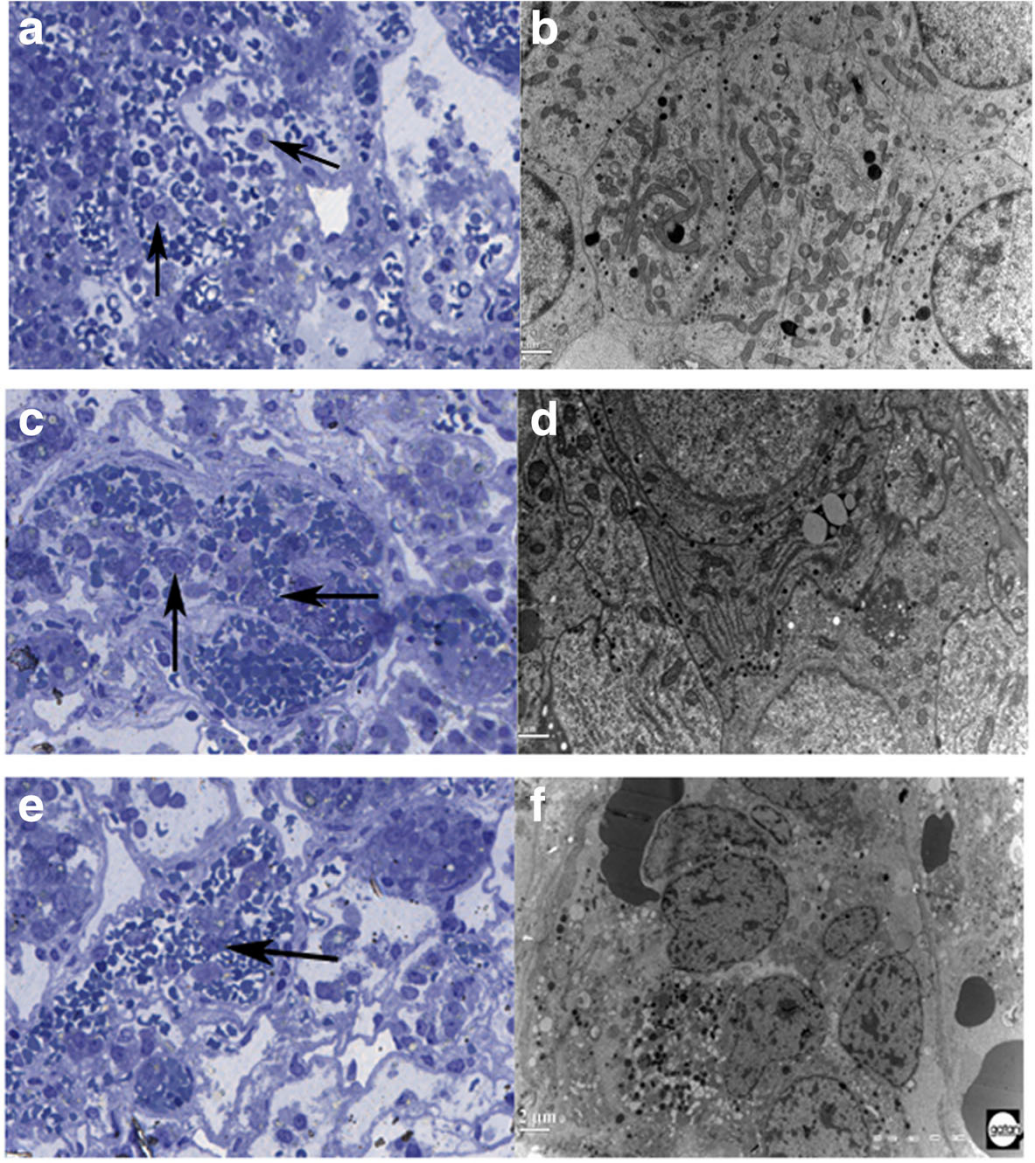
CTCs in three cases were shown. Case 1: **ab**, Case 2:**cd**, Case 3: **ef**. ACE: Tumor cells with particles in the interstitial vascular (Arrow shown) by Azure methylene blue staining.100×. BDF: TEM

A 44-year-old man was admitted to the hospital with altered visual acuity for 6 months. Magnetic resonance imaging (MRI) revealed a mass measuring 32 × 33 × 21 mm in the sellar region with cystic components. Microsurgical resection was performed by transnasal endoscopic excision of the PA. The tumor was soft and gray red with medium blood supply. On pathological examination, the tumor was negative for prolactin (PRL), growth hormone (GH), adrenocorticotrophic hormone (ACTH), follicle-stimulating hormone (FSH), luteinizing hormone (LH), and thyroid-stimulating hormone (TSH). Tumor cells with particles were detected in the interstitial vascular compartment with thickening of the basement membrane (Fig. 1a). Transmission electron microscopy (TEM): The amount of mitochondria was significantly increased in the cytoplasm of tumor cells, with small round and large irregular shaped particles (Fig. 1b).

### Case 2

A 25-year-old man was admitted to the hospital with intermittent headache, dizziness for 5 years, and decreased left eye vision for 1 year. Defects in the left temporal retina and partial nasal visual field were observed. MRI revealed a mass measuring 3 × 22 × 22 mm in the sellar region. Microsurgical resection was performed by transnasal endoscopic excision of the PA. The tumor was soft and gray red with medium blood supply. In the pathological examination, the tumor was positive for GH and PRL (scattered). Tumor cells with particles were detected in the interstitial vascular compartment with thickening of the basement membrane (Fig. 1c). TEM: Large particles and circular particles were observed in the cytoplasm of tumor cells. The rough endoplasmic reticulum was rich in lamellar assemblies and swelling of mitochondria was observed (Fig. 1d).

### Case 3

A 40-year-old woman was admitted to the hospital with headache and amenorrhea for 6 months. MRI revealed a mass measuring 20 × 20 × 15 mm in the sellar region. Microsurgical resection was performed by transnasal endoscopic excision of the PA. The tumor was soft and gray red with medium blood supply. In the pathological examination, the tumor was positive for GH and PRL (scattered). Tumor cells with particles were detected in the interstitial vascular compartment with thickening of the basement membrane (Fig. 1e). TEM: There were round or irregular particles in the cytoplasm of tumor cells. Nuclei were varied in size, with irregular shape and increased heterochromatin (Fig. 1f).

The CARE guidelines were followed when reporting these cases.

### Expression of the mesenchymal transition biomarkers β-catenin, E-cadherin, Ki-67, and p53 in PA specimens

The expression of β-catenin, E-cadherin, Ki-67, and p53 was analyzed in three cases with CTC and 24 PA specimens. The specimens were fixed in 4% buffered formalin, embedded in paraffin, and incubated with anti-β-catenin (ZSGB-BIO, China), anti-E-cadherin (Abcam, UK), anti-Ki-67 (Leica, Germany), and anti-p53 (Leica, Germany) antibodies followed by immunodetection using the two-step polymer detection system (Polink-2 plus Kit; GBI Labs, England) and visualization with 3,3′-diaminobenzidine. Five fields at 100× magnification were randomly selected.

**Fig. 2:**
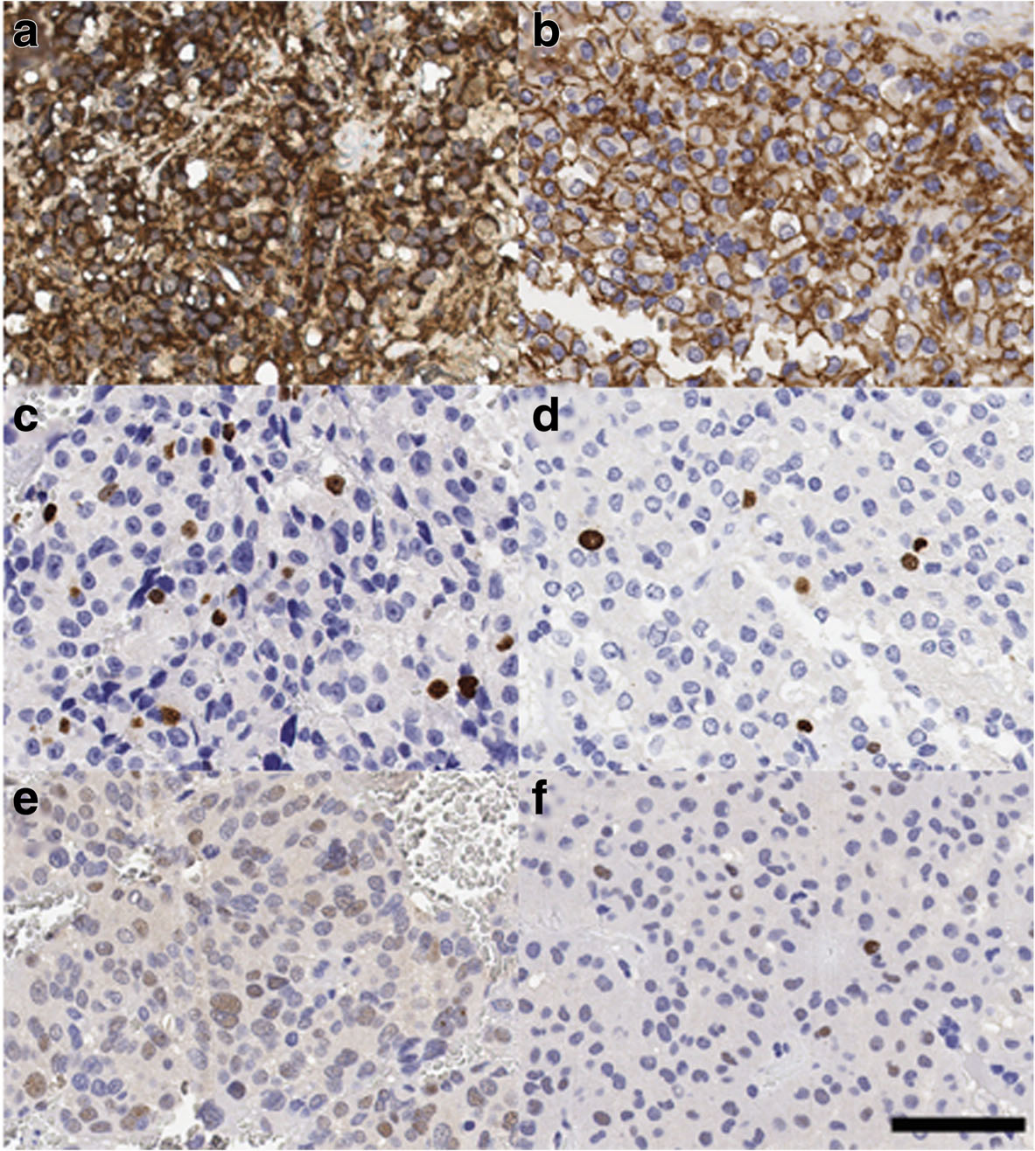
IHC analysis of the β-catenin and Ki67 expression. **a** β-catenin expression in PAs with CTCs. **b** β-catenin expression in control PAs. **c** Ki67 expression in PAs with CTCs. **d** Ki67 expression in control PAs. **e** p53 expression in PAs with CTCs. **f** p53 expression in control PAs. Bar = 60 μm

Staining scores were determined by a semi-quantitative system. The proportion of positive cells was scored as follows: 0: < 10%, 1: 10–25%, 2: 26–50%, 3: 51–75%, and 4: > 75%. Staining intensity was evaluated as follows: 0: no staining, 1: weak staining, light yellow, 2: moderate staining, yellowish brown, and 3: strong staining, brown. The sum score was determined by multiplying the positive proportion score by the intensity score. Immunohistochemical analysis showed that the β-catenin score in the three cases with CTCs was 10.5 ± 1.5 compared with 2.4 ± 0.5 in the control adenomas (Fig. 2a and b). The difference was statistically significant according to Fisher’s exact test (*p* = 0.020). The Ki-67 labeling index (Fig. 2c and d) was 2.1 ± 0.7 in the three cases with CTCs compared with 0.2 ± 0.3 in control cases (*p* = 0.043). The p53 score (Fig. 2e and f) was 4.33 ± 1.3 vs. 0.31 ± 0.17 in the controls (*p* = 0.000). The E-cadherin score did not differ significantly between the two groups (data not shown).

Molecular information obtained from the blood of cancer patients is an emerging and powerful research tool with great potential as a companion diagnostic factor for patient stratification and monitoring [12]. We detected CTCs with secretory granules in the vessels of patients with PAs. Recent experimental and clinical studies show that CTCs can be detected not only in late-stage malignant tumors with apparent distant metastases, but also in early-stage disease [13]. Furthermore, CTCs were identified as prognostic factors in several solid tumors such as colorectal, pancreatic, gastric, breast, and genitourinary cancers [14].

Pituitary carcinomas are classically defined as pituitary tumors with subcranial, brain, or systemic metastases [15]. In general, pituitary carcinomas are associated with poor prognosis as therapeutic options are limited. Whether pituitary carcinomas develop from adenomas or if they occur de novo remains unclear; in addition, the effect of hormonal subtypes on tumor aggressiveness, treatment outcomes, and prognosis has not been elucidated [16]. The present findings provide indirect evidence supporting that pituitary carcinomas develop from adenomas.

Data from a retrospective case-control study of 410 patients led to the classification of PAs according to tumor size and immunohistochemical type, and the assessment of invasion and proliferation provides prognostic information for predicting postoperative disease-free outcome or recurrence/progression status [17]. Epithelial-mesenchymal transition (EMT) is widely recognized as a key process associated with malignant transformation, and the Wnt/β-catenin pathway plays a regulatory role in EMT because of its involvement in maintaining epithelial integrity and tight adhesion junctions [18]. β-catenin, an important effector in the Wnt signaling pathway, is important for cell-cell adhesion. The E-cadherin/ β-catenin complex functions in intercellular junctions that promote cell adhesion [19]. We found that the Ki-67 LI was > 10%, and the β-catenin positivity rate was > 90% (score: 10.5 ± 1.5) in cases with CTCs. However, there was no statistically significant difference in the level of E-cadherin between the two groups. Abnormal β-catenin expression was correlated with more advanced disease stage, and as a consequence, with poor outcome in gastric cancer [20]. Nuclear E-cadherin is common in nonfunctioning PAs and a subset of growth hormone-secreting adenomas, in which it is associated with tumor size and invasion [21].

Ki-67, a marker of cellular proliferation, has been studied extensively in pituitary neoplasias [22]. It is of relevance to various clinicopathological parameters, including tumor subtype, size, invasiveness, and recurrence, as well as patient age and sex [23]. In malignant prolactinoma, the Ki-67 index of the tumor was 24.8%, and 60% of tumor cells were positive for p53 [24]. In another study, pathology revealed a high Ki-67 index in metastatic tumor tissues in two cases (24.2% and 10%) with positive p53 staining in one pituitary carcinoma [25]. In the current tumor classification of the World Health Organization, the definition of an atypical PA includes a Ki-67 labeling LI > 3% and extensive p53 positivity. In fact, PAs with Ki-67 expression ≥1.5% showed a higher recurrence risk and a worse disease-free survival compared with those with Ki-67 expression < 1.5% [26].

## Conclusion

Tumor recurrence is a significant clinical problem in PAs in which patients are asymptomatic because disseminated cells that become dormant are undetectable by clinical tools. Patients with CTCs may benefit from aggressive treatment such as radiotherapy and close surveillance.

## Abbreviations

ACTH: Adrenocorti cotrophic hormone
CTCs: Circulating tumor cells
EMT: Epithelial-mesenchymal transition
FSH: Follicle-Stimulating Hormone
GH: Growth hormone
Ki67 LI: Ki67 labelling index
LH: Luteinizing hormone
MRI: Magnetic resonance imaging
PAs: Pituitary adenomas
PRL: Prolactin
TEM: Transmission electron microscope
TSH: Thyroid-stimulating hormone
